# Changes in mental health, wellbeing and personality following ayahuasca consumption: Results of a naturalistic longitudinal study

**DOI:** 10.3389/fphar.2022.884703

**Published:** 2022-10-26

**Authors:** Daniel Perkins, Broc A. Pagni, Jerome Sarris, Paulo C. R. Barbosa, Richard Chenhall

**Affiliations:** ^1^ University of Melbourne, School of Population and Global Health, Melbourne, VIC, Australia; ^2^ Psychae Institute, Melbourne, VIC, Australia; ^3^ University of Melbourne, School of Social and Political Science, Melbourne, VIC, Australia; ^4^ Swinburne University, Centre for Mental Health, Melbourne, VIC, Australia; ^5^ Arizona State University, School of Life Sciences, Tempe, AZ, United States; ^6^ Western Sydney University, NICM Health Research Institute, Sydney, NSW, Australia; ^7^ Florey Institute for Neuroscience and Mental Health, Melbourne, VIC, Australia; ^8^ Universidade Estadual de Santa Cruz, School of Philosophy and Human Sciences, Ilheus, Bahia, Brazil

**Keywords:** ayahuasca, psychedelic therapy, depression, anxiety, alcohol use, cannabis use, self-effiacy, personality

## Abstract

**Background:** Naturalistic and placebo-controlled studies suggest ayahuasca, a potent psychedelic beverage originating from Indigenous Amazonian tradition, may improve mental health, alter personality structure, and reduce alcohol and drug intake. To better understand ayahuasca’s therapeutic potential and to identify factors that influence therapeutic efficacy, we conducted a naturalistic, longitudinal study of facilitated ayahuasca consumption in naïve participants using a comprehensive battery of self-report questionnaires.

**Materials and Methods:** Ayahuasca naive individuals registering for ayahuasca ceremonies were asked to complete a range of validated questionnaires assessing mental health, alcohol/cannabis use, relationships, personality, and connection to self and spirituality, prior to and 1 month after attending an ayahuasca ceremony. Data for two mental health measures (the DASS-21 and PANAS) and acute subjective effects *via* the MEQ-30 were also assessed 7 days post-ceremony. Repeated measures ANOVA were used to examine pre-to-post changes, and Pearson correlations explored predictors of improvement in outcomes.

**Results:** Fifty-three attendees (32 women, 21 men) completed pre and post ayahuasca assessments with 55.6% of the sample reporting a complete mystical experience based on the MEQ-30. One-month post-ayahuasca, significant reductions were identified in depression, anxiety, stress, alcohol and cannabis use, body dissociation, accepting external influence, self-alienation, impulsivity, and negative affect/emotionality. Significant increases were identified in positive mood, self-efficacy, authentic living, extraversion, agreeableness, open-mindedness, spirituality, and satisfaction with relationships. While facets of the mystical experience held little predictive validity on outcome measures, baseline traits, particularly high negative emotionality and body dissociation, and low sense of self-efficacy, robustly predicted improvements in mental health and alcohol/cannabis use, and alterations in personality structure which are linked to better mental health.

**Discussion:** This study suggests facilitated ayahuasca consumption in naïve participants may precipitate wide-ranging improvements in mental health, relationships, personality structure, and alcohol use. Associations between baseline traits and therapeutic improvements mark an important first step toward personalized, precision-based medicine and warrant randomized controlled trials to confirm and elaborate on these findings.

**Contribution Statement:** Longitudinal, observational studies and randomized clinical control trials suggest ayahuasca may exert therapeutic effects on mental health and alcohol/cannabis use, and alter personality structure. However, it is unclear if improvements are diagnosis-specific and factors that predict therapeutic gains have yet to be extensively elucidated. This longitudinal, observational study examined the effects of facilitated ayahuasca consumption in naive participants on mental health, alcohol and substance use/abuse, personality traits, relationships, and connection to self and spirituality. We found wide-ranging improvements 1-month post-treatment across these domains, and identified baseline traits which predict pre-to-post changes on primary outcome measures. Improvements were not diagnostic-specific, suggesting ayahuasca may be generally efficacious. Personality traits, body dissociation, and self-efficacy were strong predictors of therapeutic improvements, marking an important first step toward personalized, precision-based medicine. Randomized controlled trials are warranted to confirm and elaborate on these findings.

## 1 Introduction

Ayahuasca is a psychedelic plant decoction that has been consumed for centuries by Indigenous peoples in Peru, Columbia, Ecuador, and Brazil for medicinal and spiritual purposes (Luna, 2011). It contains the powerful classic psychedelic, *N,N*-dimethyltryptamine (DMT), usually from the leaves of *Psychotria viridis*, as well as several beta-carboline alkaloids, from the vine of *Banisteriopsis caapi* (McKenna et al., 1984). Recent decades have witnessed growing worldwide popularity of ayahuasca *via* expanding ayahuasca tourism in South American countries, the international expansion of Brazilian Christian-syncretic ayahuasca churches, and the growth of neo-shamanic ayahuasca ceremonies (typically for therapeutic purposes) in Western countries (Trichter, 2010; Kavenská and Simonová, 2015; Fotiou, 2020).

Mounting evidence suggests that ayahuasca and its DMT and harmala alkaloids may provide therapeutic benefit for mental health conditions, including depression, anxiety, and substance abuse disorders (Sarris et al., 2021; Uthaug et al., 2021; van Oorsouw et al., 2021; James et al., 2022). Placebo-controlled preclinical studies, as well as double-blind randomized placebo-controlled and observational naturalistic studies have reported antidepressant and anxiolytic effects (Dos Santos et al., 2016; Cameron et al., 2018; Palhano-Fontes et al., 2018; Santos da Silva et al., 2018; Almeida et al., 2019; Sarris et al., 2021; Zeifman et al., 2021). Naturalistic ayahuasca studies have also reported increases in self-compassion, and self-assurance alongside mental health improvements (Dominguez-Clave et al., 2021; Ruffell et al., 2021). A large cross-sectional study linked aspects of the subjective ayahuasca experience including self-insights with perceived improvements in depression and anxiety, suggesting that such subjective experiences play an important role in therapeutic response (Perkins et al., 2021; Sarris et al., 2021; Perkins et al., 2022).

Observational and preclinical studies also suggest that ayahuasca may reduce alcohol and other drug use (Rodrigues et al., 2021). Several studies have reported ayahuasca consumption to lower recidivism in drug-dependent adults and be a possible protective factor for alcohol and substance abuse in adolescents (Doering-Silveira et al., 2005; Fábregas et al., 2010; Barbosa et al., 2018; Rodrigues et al., 2021). Similarly, a recent large study of ayahuasca drinkers in different contexts of use reported ayahuasca consumption to be associated with increased odds of rarely or never consuming alcohol, not engaging in ‘risky drinking’, and having no recent use of a range of drugs, with these effects evident for those with and without a substance use disorder and after adjusting for religious and social group effects (Perkins et al., 2021).

Ayahuasca has also been linked to changes in personality traits (Bouso et al., 2015; Mendes Rocha et al., 2021; Weiss et al., 2021). Increases in agreeableness and openness and decreases in neuroticism have been observed, with reductions in neuroticism correlating with the subjective intensity of the mystical experience (Netzband et al., 2020). Mediators of ayahuasca’s therapeutic effects may involve changes in personality structure, as ayahuasca-induced reductions in grief, for example, have been linked to increases in acceptance and the ability to psychologically decenter (Gonzalez et al., 2021). Research to date suggests that ayahuasca may produce trait-level changes more rapidly than behavioural interventions targeting these traits (Soler et al., 2018).

Accumulating evidence suggest that ayahuasca is relatively safe and nontoxic (Gable, 2007; Barbosa et al., 2012). Cross-sectional studies have reported long-term ayahuasca drinkers to score higher on measures of well-being and life purpose, perform better on executive functioning tasks, and have lower levels of psychopathology compared to non-psychedelic users (Bouso et al., 2012; Lawn et al., 2017).

Together, these studies suggest ayahuasca may produce a range of clinically meaningful improvements. However, it is unclear if baseline individual differences predict improvements in mental health and substance use, an important step towards personalized medicine. Moreover, while research suggests ayahuasca may alter personality traits, how these changes relate to therapeutic efficacy remains to be investigated. This longitudinal, naturalistic observational study sought to examine mental health, substance use, personality traits, acute subjective effects, and relationship satisfaction outcomes after a facilitated ceremony in ayahuasca naïve adult participants. Relationships between personality traits and acute subjective effects and long-term outcomes were explored to identify predictors of therapeutic response.

Such observational research may yield more ecologically valid ayahuasca health-related data than clinical trials conducted in highly controlled settings (*cf.* Carhart-Harris et al., 2022). Less strict inclusion criteria, for example, can enable an assessment of risk factors for negative outcomes, which would otherwise be missed by the stricter criteria of double-blind clinical trials. Naturalistic longitudinal studies with new ayahuasca users can also gather “real-world” evidence of psychological and contextual variables that influence potential therapeutic benefits and risks (Pallavicini et al., 2021). The ecological validity of observational studies can provide valuable data to inform public health and drug regulation relating to the use of this Schedule I substance-containing brew in diverse therapeutic and religious/spiritual settings in the North America and elsewhere (*cf.* Bullis, 2008). Finally, the data relating to positive or negative outcomes for different conditions can inform clinical trials in the emerging field of psychedelic science.

## 2 Methods

### 2.1 Participants and design

This longitudinal observation study consisted of 53 ayahuasca naïve participants recruited *via* convenience sampling from two independent ayahuasca spiritual organisations (self-described as churches) in North America from 2019 to 2021. After successful online registration and payment at the church websites, all ayahuasca naïve attendees were emailed information about the study and an invitation to participate (*via* a survey web link) by the church organisers. The research team was not involved in recruitment, administration of ayahuasca, or conducting the ceremony. Screening criteria for ceremony participation were set by the two ayahuasca churches in the form of a questionnaire completed by all attendees. Those who were currently on medications with risk of adverse reactions (e.g., antidepressants, antipsychotics, etc) or with a diagnosed or suspected diagnosis of schizophrenia were not permitted to register. Inclusion criteria for participation in the research consisted of being an English speaker, 18 years of age or older at the survey date, and having no previous history of ayahuasca use.

Individuals who chose to participate were asked to undertake the baseline survey (BL) in the week before their first ceremony and provided written informed consent when commencing this survey. Follow-up questionnaires were requested, by email, from participants 7 days (D7) and 1-month (1 m) after the date of their first ceremony.

### 2.2 Setting

Ayahuasca was administered in a neo-shamanic mestizo-styled ceremonial setting at two independent ceremony sites between November 2018 and December 2019. At both sites participants partook in a 1-day, 2-day, or 3-day ceremony alongside 8–50 other attendees. The facilitators at each site were accompanied by 2-9 helpers–dependent on group size–who were experienced in the ceremonial ritual and were responsible for participant safety and support. Both facilitators had around 1 year in-person direct training in Peru in the Peruvian mestizo shamanic method, plus 3–9 years indirect training. Ceremonies were initiated at near or complete darkness around 9PM and lasted around 7 h. They included recorded and live traditional songs referred to as “icaros” sung in Spanish, and Quechua, as well as “New Age” spiritual songs sung in English. Participants were able to drink ayahuasca one to two times over the night and receive intranasal administration of tobacco snuff from the facilitator. Other traditional ceremonial rituals were performed, including the facilitator applying flower water on the participants hands and head and patting the chest and the head with a leaf instrument.

The ceremonial sites had single mattresses for each participant placed alongside the perimeter of a room or in a circle outside. Each attendee was provided with toilet paper and a bucket given the potential purgative effects of the beverage. Both facilitators encouraged participants to set intentions and desired outcomes for the ceremony at home prior to arrival and follow a generally healthy vegetarian diet (avoiding processed and fermented foods, dairy, yeast products, drugs and alcohol) the week before and after ceremony. No specific practices outside of the ceremonies, such as sharing circles or structured integration assistance were provided, however participants may have received informal social support from other attendees and the facilitators.

### 2.3 Measures

A range of validated mental health, wellbeing and personality instruments were selected based on the types of participant changes commonly reported in the ayahuasca literature. Demographic information was collected at baseline including sex, age, highest level of education, current labour market status, US or non-US citizenship, and previous psychedelic use.

### 2.4 Primary measures

#### 2.4.1 Mental health

Participants were asked to report lifetime mental health diagnoses, and at baseline, 7 days and 1 month we administered the DASS-21 (Depression, Anxiety and Stress Scale), a 21-item survey featuring a total score and subdomain scores for depression, anxiety, and stress (Lovibond and Lovibond, 1995), and the Positive and Negative Affect Scale (PANAS), a 10-item survey with subscale scores for positive and negative affect (Watson et al., 1988). The Short-form Health Survey (SF-12; (Maruish, 2012), a 12-item survey was administered at baseline and 1 month.

#### 2.4.2 Substance use

The World Health Organization Alcohol, Smoking, and Substance Involvement Screening Test (ASSIST), an 8-item survey which evaluates degree of substance use across different drug classes (Humeniuk et al., 2008), and two items (1. Frequency and 2. Extent of alcohol consumption) from the Alcohol Use Disorder Identification Test (AUDIT; McNeely et al., 2014), a validated 10-item survey were administered at baseline and 1 month.

#### 2.4.3 Personality traits and impulsivity

Personality traits were assessed *via* the 15-item short form Big Five Inventory-2 (BFI-2-XS) (Soto and John, 2017), and impulsivity *via* the validated 30-item Barratt Impulsivity Scale (BIS-30), comprised of behavioural and cognitive impulsivity subscales (Vasconcelos et al., 2012). All these measures were taken at baseline and 1 month.

#### 2.4.4 Acute hallucinogenic effects

Mystical Experience Questionnaire (MEQ-30) is a validated measure of spiritual/peak experiences under the influence of psychedelics, which is comprised of four dimensions: Mystical Experience, Positive Mood, Transcendence of Time/Space, and Ineffability, and also calculated as a total mystical experience score (MacLean et al., 2012). The MEQ was administered at 7 days. The score was calculated in relation to all their ceremonies undertaken in the 7-day period.

### 2.5 Secondary measures

#### 2.5.1 Relationships

Change in interpersonal relationships were assessed *via* 7-items assessing satisfaction with interpersonal relationships (Wooden et al., 2002), and relationship with nature *via* the 21-item Nature Relatedness Scale (NR; Nisbet and Zelenski, 2013).

#### 2.5.2 Connection to self and spirituality

Several instruments were used to capture change in Self connection and spirituality. The validated 10-item General Self-efficacy Scale (GSE) assesses general perceived self-efficacy (Schwarzer and Jerusalem, 2010); the 12-item Authenticity Scale assessing (AS), which contains three subscales, self-alienation, authentic living, and accepting external influence (Wood et al., 2008); and the 20-item Scale of Body Connection (SBC), which contains body awareness and bodily dissociation subscales (Price et al., 2017). The spirituality instruments utilized were the 6-item Intrinsic Spirituality Scale (ISS; Hodge, 2003), and an adapted version of the Duke University Religion Index (DUREL), which is a 5-item measure of religious involvement (Arndt and Harold, 2010). All these measures were taken at baseline and 1 month.

### 2.6 Statistical analysis

For instruments which had data available at three timepoints (baseline, 7 days, and 1-month) a repeated measures ANOVA with Bonferroni multiple comparison correction was used. Instruments with data at two timepoints were analysed using repeated measures ANOVA. Primary measures were hypothesis-driven and therefore analysed without multiple comparison correction with significance thresholds set a *p* < 0.05. Secondary measures were adjusted due to multiple comparisons by setting significance thresholds at *p* < 0.01. Violations of sphericity were corrected using Greenhouse-Geisser (DASS-Depression, DASS-Anxiety). Exploratory two-tailed Pearson correlations were performed for baseline trait measures and significant symptom change scores. Significance levels were set at *p* < 0.05 with effect sizes reported as Partial Eta Squared values. Relationships between the frequency of ayahuasca consumption 7-day post-ceremony and mental health and alcohol/substance use outcomes at 1-month were explored with Pearson correlations (*p* < 0.05) to assess potential dose-response relationships; comparisons between previous non-ayahuasca psychedelic users and nonusers and relationships with primary outcomes were explored with Pearson correlations (*p* < 0.05) to assess if previous psychedelic use impacted observed results.

## 3 Results

### 3.1 Demographics

The sample consisted of 53 ayahuasca naïve participants, including 32 females (60.4%) and 21 males (39.6%) aged between 22 and 64 (average 37.0) who have never previously used ayahuasca. Over half (52.8%) of participants had completed university level education (35.9% Bachelor, 11.3% Masters, 5.7% Doctoral degree). Around two-thirds were employed full-time (66.0%) and 17% part-time, while 11.3% listed study as their current main activity, 1.9% not working and looking for work, and 3.8% not working and not looking for work. All participants except one were residents of the United States.

Thirty participants (56.6%) reported no lifetime mental health diagnosis. Of those with a lifetime diagnosis, depression was most frequently reported by 18 individuals (34.0%), followed by an anxiety disorder (12; 22.6%), post-traumatic stress disorder (11; 20.8%), and an alcohol or drug use disorder (8; 15.1%). Three people reported ADHD, three bipolar disorder, and one person a personality disorder. The mean DASS-21 scores at baseline were 10.8 (SD 9.8) depression, 7.4 (SD 7.5) anxiety, and 15.1 (SD 8.9) stress, with 42%, 40%, and 42% of participants respectively being ranked outside of the normal range on these scales.

### 3.2 Mental health

**FIGURE 1:**
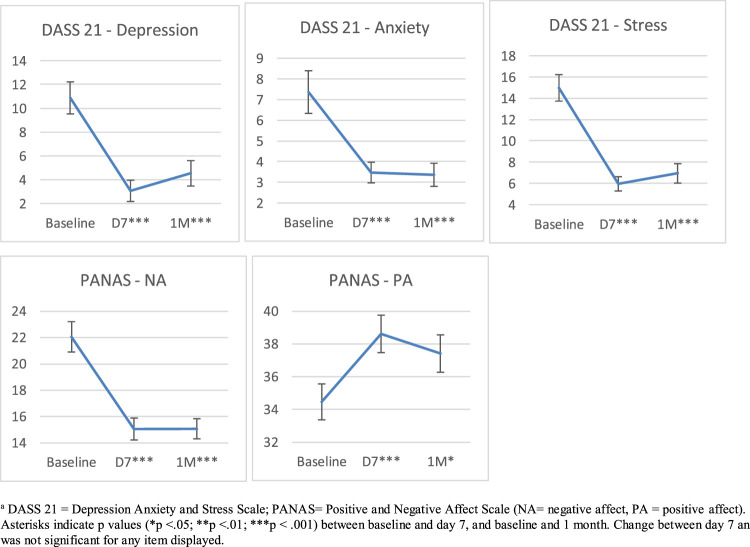
DASS 21 and PANAS mean scores at baseline, day seven (D7), and 1 month (1 M)^a^.

Significant main effect of time across baseline, 7 days, and 1 month on the DASS-21 were detected for total scores, indicating decreases in anxiety, stress, and depression (Table 1; Figure 1). Changes on the PANAS from baseline, 7 days, and 1 month indicated increases in positive affect and decreases in negative affect (Table 1; Figure 1). Pairwise comparisons on all DASS-21 and PANAS scales indicated significant changes over time from baseline to 7 d and baseline to 1 m, but no significant changes from 7 d to 1 m after Bonferroni correction (Table 1). Significant BL to 1 m change was also identified on the SF-12 mental composite score, but not the physical health composite score (PCS) (Table 1).

**TABLE 1:** Primary outcomes repeated measures at baseline, day seven (where available), and 1 month. Values are given as means (standard deviations in brackets).

Measures	n	Baseline	Day 7	*p*-value BL-D7	1 Month	F-value BL-D7-1 m or BL-1M	*p*-value BL-1m
*MENTAL HEALTH*							
DASS-21^a^							
Depression**^b^**	53	10.87 (9.80)	3.06 (6.49)^***^	<0.001	4.53 (7.77)***	20.01	<0.001
Anxiety**^b^**	53	7.36 (7.50)	3.47 (3.62)^***^	<0.001	3.36 (4.07)***	14.28	0.001
Stress	53	14.98 (9.06)	5.96 (4.88)^***^	<0.001	6.94 (6.65)***	41.02	<0.001
Total score	53	33.21 (23.51)	12.49 (11.78)^***^	<0.001	14.83 (15.88)***	34.07	<0.001
PANAS^a^							
Negative affect	53	22.06 (8.40)	15.06 (6.05)^***^	<0.001	15.08 (5.57)***	27.64	<0.001
Positive affect	53	34.47 (7.99)	38.62 (8.34)^***^	<0.001	37.42 (8.32)*	9.83	0.035
SF-12 (Short-form Health Survey)							
Mental health summary (MCS)	53	39.62 (12.63)			49.62 (9.56)***	33.66	<0.001
Physical health summary (PCS)	53	52.42 (8.43)			52.34 (7.39)	Ns	0.934
*SUBSTANCE USE*							
ASSIST V3							
Alcohol	34	9.76 (4.65)			8.26 (3.57)**	13.14	0.001
Cannabis	31	15.13 (4.97)			12.84 (4.43)**	14.68	0.001
AUDIT							
Frequency of Alcohol Consumption	53	1.75 (1.34)			1.30 (1.22)**	11.50	0.001
Average Alcoholic Beverages	35	0.66 (1.08)			0.46 (0.89)	2.02	0.165
Frequency of Binge Drinking	35	0.94 (1.00)			0.80 (0.933)#	2.93	0.096
*PERSONALITY*							
BFI-2-XS							
Extraversion	53	9.87 (2.73)			10.49 (2.47)*	4.85	0.032
Agreeableness	52	11.83 (2.13)			12.19 (2.13)*	4.02	0.050
Conscientiousness	52	10.63 (2.45)			11.15 (2.75)#	3.27	0.077
Negative Emotionality	53	8.89 (3.72)			6.96 (3.57)***	20.11	<0.001
Open-Mindedness	53	12.30 (2.44)			13.00 (1.75)**	7.85	0.007

^a^ Bonferroni correction performed.

^b^ Greenhouse-Geisser correction performed.

Asterisks indicate *p* values: *p < .05; **p < .01; ***p < .001.

### 3.3 Alcohol and cannabis use

Significant reductions in substance use from baseline to 1 m were observed for alcohol and cannabis use according to the ASSIST (Table 1; Figure 1). Frequency of alcohol consumption was also reduced at 1 m according to Item 1 on the AUDIT alongside a trend toward a reduction in the frequency of binge episodes (defined as ≥ 5 beverages (Table 1). However, no changes in the average number of alcoholic beverages consumed were found (Table 1).

### 3.4 Personality

Significant changes on BFI traits “Extraversion”, “Agreeableness”, “Negative Emotionality”, and “Open-mindedness”, but not “Conscientiousness” were found (Table 1).

## 4 Secondary measures

### 4.1 Connection to self and spirituality

Significant increases in self-efficacy, the Authenticity subscale of “Self-Alienation”, and the Scale of Body Connection subscale “Body Dissociation” were observed 1 m post-ayahuasca. The other two Authenticity subscales, “Accepting External Influence”, and “Authentic Living”, were trending towards significant, while no change on the SBC subscale “Body Awareness” was observed (Table 2). Significant increases in spirituality were detected *via* the Intrinsic Spirituality Scale, but no changes in religiosity were apparent *via* the DUREL (Table 2).

**TABLE 2:** Secondary outcomes repeated measures at baseline, day seven (where available), and 1 month. Values are given as means (standard deviations in brackets).

Measure	n	Baseline	1 Month	F-value BL-1m	*p*-value BL-1m
General Self-Efficacy ^b^	52	32.02	34.25***	16.55	<0.001
Scale of Body Connection^b^					
Body awareness	51	2.75 (0.65)	2.73 (0.65)	0.03	0.864
Body dissociation	51	1.17 (0.73)	0.82 (0.51)**	10.91	0.002
Authenticity Scale ^b^					
Authentic living	53	22.62 (4.07)	23.53 (4.13)#	4.97	0.023
External influence	53	12.72 (6.66)	10.94 (5.40)#	16.04	0.030
Self-alienation	52	11.73 (6.53)	8.27 (4.63)***	5.51	<0.001
Nature Relatedness Scale ^b^	53	4.13 (0.50)	4.19 (0.54)	ns	0.239
Self	53	4.23 (0.60)	4.31 (0.63)	ns	0.191
Perspective	53	4.04 (0.60)	4.03 (0.64)	ns	0.965
Experience	53	4.11 (0.69)	4.20 (0.74)	ns	0.229
Intrinsic Spirituality Scale ^b^	53	7.00 (2.48)	7.85 (2.11)**	10.33	0.002
DUREL (religious meetings) ^b^	53	1.25 (0.76)	1.28 (0.79)	ns	0.485
DUREL (private religious) ^b^	52	1.87 (1.70)	2.04 (1.80)	ns	0.430
Spiritual activities ^b^	53	5.02 (1.81)	5.26 (1.40)	ns	0.212
Relationship satisfaction ^b^					
Partner	33	6.15 (2.88)	6.61 (2.60)	ns	0.249
Friends	52	6.40 (2.75)	7.12 (2.28)#	4.29	0.043
Children	21	7.38 (2.50)	8.05 (1.99)#	4.59	0.045
Parents	48	6.23 (2.77)	6.90 (2.86)#	4.07	0.050
Partners parents	24	5.83 (2.97)	6.88 (2.66)	2.95	0.099
Other family	50	6.02 (2.59)	6.54 (2.34)	2.98	0.091
Former Partner	38	5.08 (3.23)	5.89 (3.52)	ns	0.225
Barratt Impulsiveness Scale ^b^	53	64.90 (10.51)	63.03 (10.36)#	4.03	0.050
Cognitive	53	40.73 (7.05)	39.32 (7.47)#	4.48	0.039
Behavioural	53	31.36 (6.60)	30.10 (6.26)#	4.69	0.050

Asterisks indicate *p* values: *p < .01; **p < .0.005; ***p < .001^#^ < .0.05.

### 4.2 Relationships

None of the relationship satisfaction items showed significant change, however three of these items (relationships with friends, children, and parents) were suggestive of improvement, and would be significant at the *p* < 0.05 level (Table 2). No changes were observed on the Nature Relatedness Scale.

### 4.3 Impulsivity

Neither the impulsivity total score (BIS11) or the subscales, “Cognitive Impulsivity” and “Behavioural Impulsivity”, changed significantly at 1-month, however, these were also trending toward significance (Table 2).

### 4.4 Change in mental health by lifetime mental health diagnosis

Changes on DASS total, MCS12, and PANAS-NA were compared between participants with and without lifetime mental health diagnoses, revealing that both groups showed improvements across these three mental health measures (Table 3). In addition, analysis by lifetime mental health diagnoses, showed statistically significant improvements in mental health for participants with ADHD, anxiety, alcohol and drug use, depression, and PTSD (Table 3).

**TABLE 3:** Relationships between lifetime (LT) diagnostic group and mental health improvements from repeated measures at baseline and 1 month^a^.

		DASS total		MCS12		PANAS NA		
		Baseline	1 month	Baseline	1 month	Baseline	1 month
No LT MH diagnosis	31	27.0 (20.7)	12.2 (11.6)^***^	42.8 (11.2)	50.8 (8.5)^**^	20.6 (7.4)	14.4 (4.8)^***^
LT MH diagnosis	24	41.4 (24.5)	21.8 (26.1)^**^	35.0 (12.3)	47.3 (11.4)^***^	24.5 (9.3)	17.6 (13.9)^**^
ADHD	3	35.3 (18.0)	12.7 (9.0)^*^	40.0 (17.1)	49.7 (9.2)	26.3 (5.0)	14 (3.6)^*^
Anxiety disorder	13	49.8 (22.2)	26.9 (26.5)^*^	32.0 (11.0)	44.9 (11.3)^***^	26.6 (8.6)	20.7 (9.3)^*^
Alcohol and Drug Use Disorder	9	42.0 (22)	24.4 (31.3)	31.9 (13.9)	46.9 (12.2)^***^	25.4 (9.6)	17.6 (10.7)^*^
Bipolar	3	46.7 (27.7)	22 (14.0)^#^	31.2 (8.0)	35.9 (8.1)	25.7 (8.4)	20 (7.2)
Depression	19	47.2 (23.2)	23.9 (28.8)^**^	31.3 (10.4)	45.0 (11.4)^***^	26.3 (9.2)	18.3 (9.5)^**^
PTSD	11	41.5 (23.2)	23.8 (24.5)^#^	35.9 (12.6)	45.3 (10.9)^#^	24.5 (9.3)	17.5 (6.7)^#^

Asterisks indicate *p* values: *p < .01; **p < .0.005; ***p < .001 # < .0.05.

^a^ Values are given as means (standard deviations in brackets); LT MH, life time mental health; ADHD, attention hyperactivity disorder.

### 4.5 Exploratory predictors of therapeutic improvements

**FIGURE 2:**
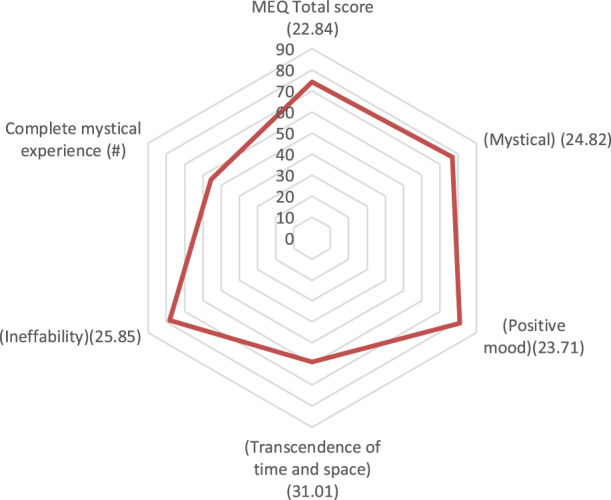
Total MEQ score and sub-scale scores (% of maximum), and proportion of people experiencing a full mystical experience (SD in brackets).

We investigated correlations between facets of the mystical experience and baseline to 1 month change scores for behavioural measures showing significant pre-to-post changes. The only significant correlations detected were on the MEQ subscale “Ineffability” with changes on the GSE, “Positive mood” with AS “Authentic Living”, and “Transcendence of Space/Time” with changes on Relationship Satisfaction with parents. Trending associations were also detected between MEQ total scores and AS “Authentic Living” (Table 4; Figure 2). Spearman Rho’s were also computed for correlations between MEQ scores and primary outcomes and results were consistent with the aforementioned Pearson correlations.

**TABLE 4:** Pearson’s correlations between baseline traits or MEQ facets and 1-month behavioural change scores.

		Change scores at 1-month																				
		PANAS- P	PANAS—N	ISS	As—Aut. Living	As—Ext. Influ	As—Self-aliena	BFI –Extrav	BFI -neg emot -	BFI- op mind -	SBC-BD	GSE	Rs - parents	SF-12 MCS	DASS anx	DASS stress	DASS depr	DASS total	ASSIST alcohol	ASSIST cannabis	AUDIT #1	
Extraversion	*r* =	-0.14	0.24	-0.29	-0.17	0.22	0.31#	0.50^***^	0.29#	-0.33#	0.09	-0.40^**^	0.04	-0.16	0.14	0.25	0.19	0.23	0.05	0.29	0.15	
Agreeableness	r =	0.01	0.10	−0.14	−0.01	0.08	0.23	-0.26	0.05	−0.12	0.25	−0.21	0.26	−0.09	0.33#	0.22	0.10	0.23	-0.22	0.06	−0.04	
Conscientiousness	r =	−0.30#	**0.46** ^***^	−0.22	0.05	0.30#	0.24	-0.13	0.08	-0.06	0.17	−0.08	0.02	−**0.44** ^***^	0.27	0.12	0.34#	0.29#	0.45*	0.20	0.21	
Negative Emotionality	r =	0.17	−**0.49** ^***^	0.20	0.24	−**0.39** ^**^	−**0.37** ^*^	0.30#	−**0.47** ^***^	0.22	−0.30	**0.40** ^**^	0.13	**0.46** ^**^	−**0.47** ^***^	−**0.46** ^**^	−**0.37** ^*^	−**0.49** ^***^	−0.35#	−0.42#	−0.14	
Open-Mindedness	r =	-−0.12	0.22	−0.32#	-0.25	0.09	0.29#	-0.34#	0.27	−**0.70** ^***^	**0.40** ^**^	−0.34#	0.03	−0.23	0.17	0.20	0.28	0.26	−0.04	0.42#	0.16	
SCB—Body Awareness	r =	-0.18	0.22	-0.26	-0.24	0.10	0.25	-0.22	-0.07	**-0.37** ^*^	0.04	-0.12	-0.01	-0.20	0.00	-0.03	0.13	0.05	−0.27	−0.06	0.15	
SCB—Body Disconnection	r =	0.24	**-0.54** ^***^	0.23	0.27	-0.27	**-0.54** ^***^	0.31#	**-0.44** ^**^	**0.36** ^*^	**-0.77** ^***^	0.26	-0.14	**0.37** ^*^	**-0.63** ^***^	−**0.46** ^**^	−**0.47** ^***^	**-0.59** ^***^	−0.12	−0.32	0.03	
GSE	r =	-0.25	**0.37** ^*^	-0.31	-0.21	0.15	**0.39** ^*^	-0.34	0.24	**-0.52** ^***^	0.25	−**0.60** ^***^	0.08	**-0.39** ^**^	**0.41** ^**^	**0.41** ^**^	**0.44** ^**^	**0.49** ^***^	0.18	0.25	0.21	
MEQ—Mystical	r =	0.13	-0.05	-0.06	0.21	-0.01	-0.13	0.06	-0.11	−0.13	−0.17	0.19	0.08	0.11	-0.06	-0.06	0.05	-0.02	-0.09	-0.09	0.14	
MEQ -Positive mood	r =	0.19	−0.08	-0.06	0.31#	-0.16	−0.22	0.08	−0.18	−0.07	-0.22	0.20	0.06	0.16	-0.04	-0.04	-0.05	-0.05	-0.19	-0.19	0.22	
MEQ—Space/time	r =	-0.04	−0.02	−0.04	0.14	0.00	0.01	-0.15	-0.11	-0.03	-0.15	0.04	0.29	0.00	0.06	0.06	0.19	0.13	-0.22	-0.01	0.14	
MEQ—Ineffability	r =	0.08	0.02	0.01	0.21	-0.12	-0.13	0.09	-0.04	0.00	-0.13	0.307#	0.14	0.10	-0.02	0.03	0.11	0.06	-0.09	-0.10	0.12	
MEQ total	r =	0.11	-0.05	-0.06	0.24#	-0.05	-0.13	0.02	-0.13	-0.09	-0.20	0.19	0.15	0.11	-0.03	-0.02	0.08	0.02	-0.16	-0.10	0.17	
# of sessions	R =	0.02	-0.12	0.28#	0.15	-0.10	-0.16	0.18	-0.09	0.16	-0.30#	0.02	-0.08	0.05	-0.09	-0.10	-0.18	-0.15	-0.04	-0.11	0.08	

Asterisks indicate *p* values: *p < .01; **p <.005; ***p < .001^#^ < .0.05.

*p* < 0.01.

Additionally, in an exploratory correlation analysis we investigated relationships between baseline personality traits and pre-to-post behavioural changes which yielded numerous strong predictors of mental health and substance use outcomes. Baseline personality traits from the Big Five Inventory were associated with changes on the PANAS, ISS, AS, BFI, SF-12, GSE, MCS, DAS, and ASSIST; the personality trait ‘negative emotionality’ held the most robust predictive power across a range of outcomes measures, with individuals showing greater negative emotionality at baseline demonstrating greater improvement from ayahuasca on mental health, personality-related, and substance use measures (Table 4). Moreover, baseline levels of ‘body dissociation’, a subscale on the SBC and the GSE also held strong predictive power. Those who had high degree of body dissociation and lower sense of self-efficacy demonstrated more dramatic improvements on outcome measures. Interestingly, the direction of significant correlations were identical for negative emotionality and body dissociation, and inverse for self-efficacy (Table 4). Exploratory correlations between both the frequency of ayahuasca consumption at 7-day post-ceremony and prior psychedelic use, and 1-month primary outcomes pertaining to mental health and alcohol/substance use were nonsignificant (Supplemental Table S1).

^a^DASS 21 = Depression Anxiety and Stress Scale; PANAS = Positive and Negative Affect Scale (NA = negative affect, PA = positive affect). Asterisks indicate *p* values (*p < 0.05; **p < 0.01; ***p < 0.001) between baseline and day 7, and baseline and 1 month. Change between day 7 and 1 month was not significant for any item displayed.

## 5 Discussion

Following encouraging initial data from human and animal studies with ayahuasca, there is growing interest in the potential medical use of this substance for the treatment of mental health conditions. Our findings relating to mental health effects for individuals with and without a lifetime mental health diagnosis are in-line with reports of depression, anxiety, and stress reductions in placebo-controlled studies (Palhano-Fontes et al., 2018), observational studies with follow-up periods up to 6 months (Barbosa et al., 2005; Barbosa et al., 2009; Jiménez-Garrido et al., 2020; Ruffell et al., 2021), and cross-sectional studies comparing ayahuasca users to non-users (Kaasik and Kreegipuu, 2020; Sarris et al., 2021). Participants also reported less problematic alcohol and cannabis use 1-month post-ceremony, corroborated by decreased frequency of alcohol use and a trend toward decreased alcohol binges. These findings represent the first longitudinal assessment of ayahuasca’s effects on alcohol consumption in a moderately-sized sample (n∼34). A small observational study (*n* = 12) found reductions in alcohol, tobacco, and cocaine use after ayahuasca with no changes in cannabis use (Thomas et al., 2013). This study marks the first to detect significant changes in cannabis consumption where other studies have been underpowered to detect changes in cannabis (Thomas and colleagues (2013). These findings are in-line with cross-sectional reports suggesting that ayahuasca consumption is negatively associated with alcohol and drug use, however it is still uncear if ayahuasca may reduce intake of other drugs of abuse, such as amphetamines and opiates (Halpern et al., 2008; Fábregas et al., 2010; Rodrigues et al., 2021; Perkins et al., 2022).

Consistent with previous studies we detected changes in the personality traits agreeableness and open-mindedness (Netzband et al., 2020; Kiraga et al., 2021; Weiss et al., 2021). However, a novel finding was of increases in extraversion, which although reported with psilocybin has not previously been reported with ayahuasca (Erritzoe et al., 2018). Further, decreases in negative emotionality and trending increases in conscientiousness, implicating all 5 BFI traits, suggests ayahuasca broadly influences personality structure, some of which (e.g., agreeableness) are thought to remain relatively stable throughout adult life (Specht, 2017). Previous work has found associations between the Big Five personality trait ‘neuroticism’ and psychopathology, and traits ‘extraversion’ and ‘agreeableness’ with positive mental health (Lamers et al., 2012). We propose the ayahuasca-induced changes observed in personality structure may reflect resilience toward psychopathology and improved mental health. While this study did not specifically examine predictors of personality change (Weiss et al., 2021), reported that baseline personality traits, acute experiences, ceremonial elements, and purgative experiences moderate changes in personality traits; this is consistent with our results, showing baseline personality traits predict ayahuasca attributed personality trait changes. Given this, future work may utilise mediation/moderation analyses to better understand how the ayahuasca experience and acute effects have enduring effects on personality traits and how these changes may contribute to mental health improvements. Previous cross-sectional work found that members of the UDV ayahuasca church have a greater propensity toward self-reflection *versus* impulsivity (McKenna, 2004). Although we found no significant change in behavioural and cognitive impulsivity, the trend in both measures warrants future investigation.

Previous cross-cultural studies have noted improvements in relationships (Kaasik and Kreegipuu, 2020); and again we found a trend towards significance for improvements in relationships with friends, parents, and children.

This study also included a range of novel measures intended to evaluate change in self-connection, most of which identified significant positive change. We provide the first evidence of improvements in general self-efficacy, a construct negatively associated with the likelihood of suffering from mental illness (Andersson et al., 2014). We found increases in personal authenticity, which is related to self-esteem and psychological well-being (Wood et al., 2008). Moreover, reductions in body dissociation (but not increases in body awareness) were identified, suggesting a longer-term effect of the enhanced somatic awareness and interoception known to occur with the acute ayahuasca experience (Shanon, 2002; Espinoza, 2014; Kaufman, 2015). Neurologically, ayahuasca activates brain areas involved in somatic awareness, emotional states, and arousal, namely the insular and anterior cingulate cortices and amygdala, offering potential neural correlates of the observed behavioural changes (Riba et al., 2006). Importantly, ayahuasca-induced alterations to body dissociation, but not body awareness, may expand our understanding of the complex role of the mind-body connection. Together, our findings suggest personal relationships, self-efficacy, personal authenticity, and body awareness may be important variables to consider in the context of transformative and healing psychedelic experiences.

Previous longitudinal and cross-sectional studies have showed concomitant augmentation of spirituality and religiousness, while the present study identified increases in spirituality in the absence of changes in religious practices (Bouso et al., 2012; Gonzalez et al., 2021). Lack of change in religious practices may be attributed to differences in instrument selection or in sampling differences between studies. The DUREL used in this study consists of questions pertaining to frequency of religious practices while the WHO Quality of Life Spirituality, Religiousness, and Personal Beliefs scale employed by Gonzalez and colleagues (2021) consists of questions pertaining to quality of life, personality traits, and attitudes/beliefs. Additionally, our sample was collected from a neo-shamanic ceremony setting, whereas other study samples may have consisted of members with a more religious background.

Facets of the mystical experience, as measured *via* the MEQ, have been reported to predict psilocybin-occasioned improvements in mental health, however such an association was not present in this study (Roseman et al., 2018), a finding consistent with other ayahuasca research utilising the MEQ (Palhano-Fontes et al., 2018; Ruffell et al., 2021). Since only ∼50% of our sample achieved a “complete mystical experience”, it is possible the analysis was underpowered to detect MEQ-outcome relationships. Alternatively, the MEQ may not be an optimal instrument for detecting ayahuasca induced spiritual experience, as other ayahuasca studies using alternative instruments have reported consistent associations between subjective spiritual experience and improvements in mental health, wellbeing and alcohol and drug use (Sarris et al., 2021; Perkins et al., 2022). Other aspects of the subjective experience not measured in this study, including the number of personal self-insights gained and level of extreme fear have also been reported to be associated with ayahuasca’s therapeutic outcomes (Perkins et al., 2021).

This study also identified new predictors of mental health and addiction-related improvements. Instead of subjective effects, based on the MEQ, predicting therapeutic outcomes, baseline personal characteristics pertaining to personality traits, general self-efficacy, and body dissociation at baseline strongly predicted improvements in mental health and alcohol and cannabis misuse/abuse. In our study, participants who had higher negative emotionality and body dissociation and lower self-efficacy at baseline demonstrated larger improvements on mental health outcomes. Upon close examination, the directions of correlations were identical for negative emotionality and body dissociation, and inverse that of self-efficacy for all significant behavioural change associations. The overall relationship between baseline predictors and outcomes measures suggests those who have greater psychopathological indications on these measures may be preferred candidates for therapeutic effects. The consistency of these predictors across a variety of measures fosters confidence, however the effects need to be replicated in randomised clinical trials. While others have established links between personality traits and acute effects (e.g., neuroticism associated with unpleasant/anxious experiences, trail-level ‘absorption’ associated with enjoyable psychedelic experiences, and openness and optimism associated with acute psychedelic effects; (Studerus et al., 2012; Smigielski et al., 2019; Studerus et al., 2021), this study is the first to characterize the influence personality factors have on ayahuasca-attributed improvements in mental health and alcohol/cannabis use. Moreover, these findings mark a valuable first step toward developing the personalized application of psychedelic medicine.

Therapeutic mechanisms of ayahuasca likely range from the molecular to the psychological: ayahuasca administration has been shown to up-regulate serotonin reuptake transporters in blood platelets which are downregulated in alcoholism (Callaway et al., 1994; Storvik et al., 2007). Both DMT and the beta-carbolines found in ayahuasca have been linked to physiological and psychological markers of improved mental health (Samoylenko et al., 2010). For example, DMT and the three main alkaloids of ayahuasca–namely, harmine, tetrahydroharmine, harmaline, and the metabolite harmol–stimulate neurogenesis, spinogenesis, and synaptogenesis, providing a potential biological basis for some of ayahuasca’s therapeutic effects ((Morales-García et al., 2017; Ly et al., 2018; Morales-Garcia et al., 2020). Moreover, antidepressant effects correlate with brain-derived neurotrophic factor (BDNF) concentrations 48-h after administration of a single dose of ayahuasca with enduring antidepressant effects 7 days after administration (Palhano-Fontes et al., 2018; Almeida et al., 2019). On a systems level, ayahuasca modulates the default mode network (DMN), theorized to underlie the neuropathology of depression and anxiety and support the sense of self. Reorganization of the structural and functional architecture of the DMN may mediate clinically relevant improvements across various psychiatric and substance use disorders (Palhano-Fontes et al., 2015). Finally, personality trait changes appear to drive some of ayahuasca’s therapeutic effects, including reductions in alcohol and substance use, which offers novel psychological mechanisms outside of the traditional addiction framework centred around impulsivity and novelty seeking (Ismael Eduardo Apud, 2020). Further, associations between ayahuasca use and personality traits have been correlated with cortical thickness in the posterior cingulate cortex, suggesting ayahuasca-induced structural DMN changes may support alterations to personality (Bouso et al., 2015). Future work should seek to elucidate the inter-relationships between genetics, epigenetic, biochemistry, and network dynamics in the context of psychedelic medicine.

## 6 Limitations

There are important limitations in this study to acknowledge, most notably the absence of an active comparison group with which to compare findings. A placebo-controlled naturalistic study and large cross-sectional ayahuasca study both noted significant therapeutic effects associated with non-pharmacological factors (e.g., ceremony, community, etc), which are likely to also contribute to changes identified in our sample (Perkins et al., 2021; Uthaug et al., 2021). However, similar to reports from Griffiths and colleagues studying psilocybin, ayahuasca users have reported their experiences to be among the most important of their lives (Dos Santos et al., 2016) (Perkins et al., 2021).

Further limitations include the modest sample size, use of self-report measures, which are at greater risk of participant biases, lack of data on participant expectations, and slight variations in the day of response. Future work could include neuropsychological evaluations to corroborate self- and informant-report measures. However, we note that previous work in pharmacotherapies for depression suggests that objective measures capture larger effect sizes relative to less objective measures such as the self-report surveys used in this study (Joffe et al., 1996). Additionally, our use of an extensive mental health battery provides assurance of broad range improvements across multiple domains including mood/affect, social relationships, Self-connection, impulse control, and spirituality.

While we enrolled only participants naive to ayahuasca consumption, some participants reported previous psychedelic use (e.g., psilocybin, LSD, ketamine, etc) which may influence outcomes compared to individuals with no prior psychedelic experience and who are non-ayahuasca naïve. However, previous psychedelic use was not associated with behaviour change in this study, suggesting both psychedelic-naïve and psychedelic-experienced participants may benefit from ayahuasca. Additionally, chemical analysis of the ayahuasca brew was not performed, limiting knowledge of psychoactive concentrations and ratios and dose effects. The volume of ayahuasca liquid administered was generally consistent, although was adjusted in some cases such as very high/low weight or more acute mental health symptoms. Exploratory correlation analyses showed no relationship between the number of dosing sessions and mental health and alcohol/marijuana use outcomes, which should be interpreted with caution since the study was not poised to address dose response questions. Long-term follow-up is also necessary to assess the durability of treatment effects identified. Sustained improvements have been reported 6-month posttreatment in one study, and a large cross-sectional study identified little diminishing of reported improvements in mental health or reductions in drug and alcohol use over time (Jiménez-Garrido et al., 2020; Ruffell et al., 2021). However, another study found that at a 4–7-year follow-up, treatment gains from ayahuasca were lost after a single administration, suggesting repeated administration may be necessary (dos Santos et al., 2016). It would also be useful for ayahuasca research to examine treatment trajectories for specific diagnostic groups to develop long-term treatment plans.

## 7 Conclusion

In this naturalistic observational study of facilitated ayahuasca consumption in naïve participants, we identified improvements on a myriad of mental health and wellbeing measures, in addition to changes in personality structure. Our mental health results are in congruence with previous research, and we also report novel findings relating to change in self-beliefs, self-connection, and impulsivity, in addition to predictors of therapeutic response to ayahuasca identifying a range of factors that should be considered in the context of mental health treatment. While the findings are preliminary and require confirmation using randomised clinical trial designs, they mark a useful step towards developing personalised medicine approaches for the treatment of complex psychiatric conditions. Moreover, the detection of therapeutic improvements on novel instruments warrants consideration of these measures in more rigorous designs.

Finally, we note that while studies conducted in laboratory and hospital settings provide excellent internal validity, it is also important to understand therapeutic changes as a function of the naturalistic ceremonial settings in which these practices evolved. Such contexts encompass a radically different worldview than Western medical approaches and may provide new insights to enable enhanced clinical treatments.

## Data Availability

The datasets presented in this article are not readily available because ethics approval was for data access by members of the research team only. Requests to access the datasets should be directed to Dr Daniel Perkins, d.perkins@unimelb.edu.au.
