# Supplementary material for: Changes in mental health, wellbeing and personality following ayahuasca consumption: Results of a naturalistic longitudinal study
Source: Front Pharmacol. 2022 Oct 26;13:884703. doi: 10.3389/fphar.2022.884703 (PMC9643165; doi:10.3389/fphar.2022.884703)
Supplement: Supplementary file 1 [file Presentation1.pptx]

## Slide 1
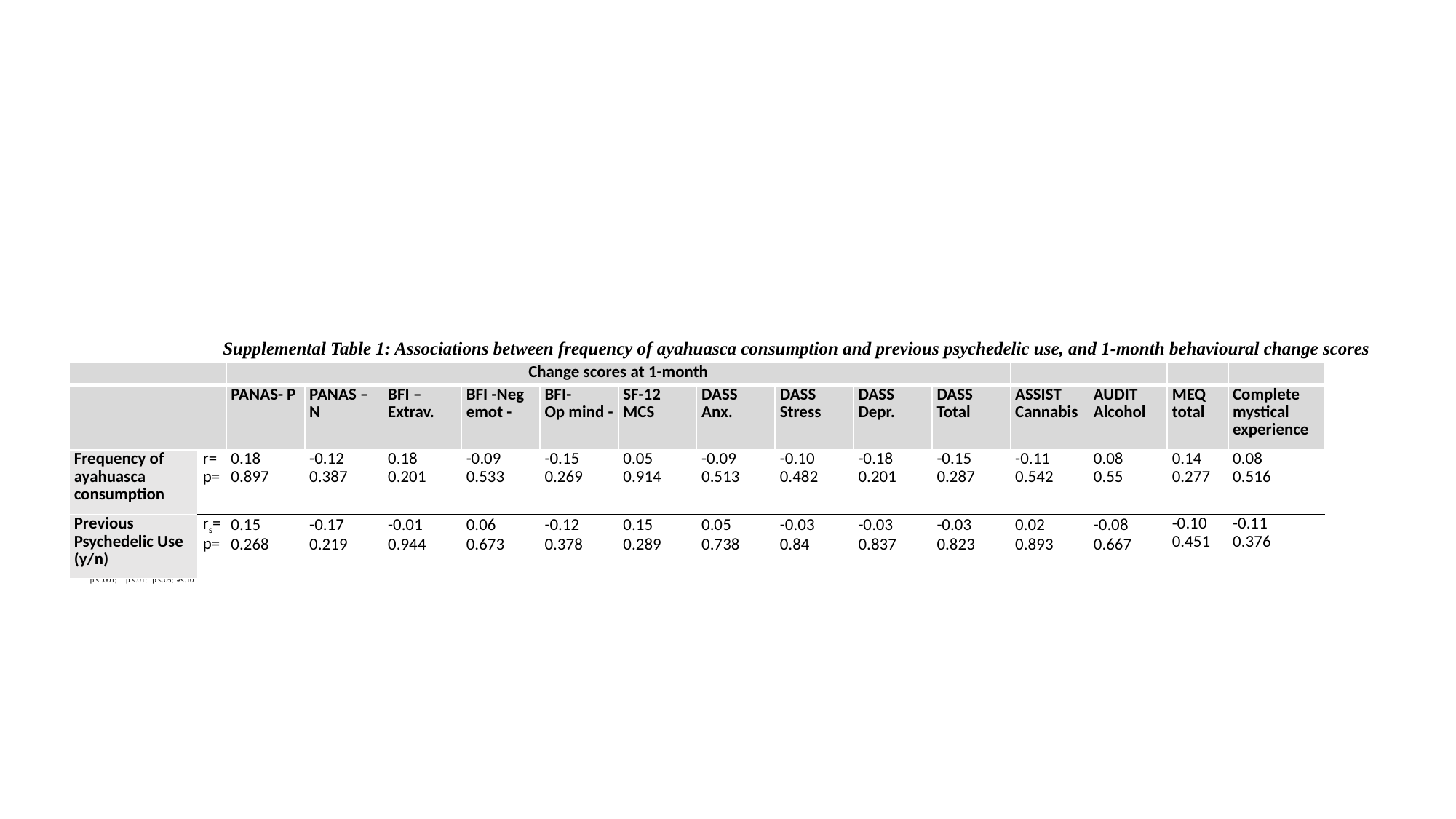

Supplemental Table 1: Associations between frequency of ayahuasca consumption and previous psychedelic use, and 1-month behavioural change scores
| | | Change scores at 1-month | | | | | | | | | | | | | |
| --- | --- | --- | --- | --- | --- | --- | --- | --- | --- | --- | --- | --- | --- | --- | --- |
| | | PANAS- P | PANAS – N | BFI –Extrav. | BFI -Neg emot - | BFI- Op mind - | SF-12 MCS | DASS Anx. | DASS Stress | DASS Depr. | DASS Total | ASSIST Cannabis | AUDIT Alcohol | MEQ total | Complete mystical experience |
| Frequency of ayahuasca consumption | r= p= | 0.18 0.897 | -0.12 0.387 | 0.18 0.201 | -0.09 0.533 | -0.15 0.269 | 0.05 0.914 | -0.09 0.513 | -0.10 0.482 | -0.18 0.201 | -0.15 0.287 | -0.11 0.542 | 0.08 0.55 | 0.14 0.277 | 0.08 0.516 |
| Previous Psychedelic Use (y/n) | rs= p= | 0.15 0.268 | -0.17 0.219 | -0.01 0.944 | 0.06 0.673 | -0.12 0.378 | 0.15 0.289 | 0.05 0.738 | -0.03 0.84 | -0.03 0.837 | -0.03 0.823 | 0.02 0.893 | -0.08 0.667 | -0.10 0.451 | -0.11 0.376 |
**p < .001; **p <.01; *p <.05; #<.10
